# Mouse model of HDM induced airway inflammation + influenza or RSV. Effects of steroid

**DOI:** 10.1186/1476-9255-10-S1-P22

**Published:** 2013-08-14

**Authors:** Dave Lamb, Christina Muir, Debbie Chappell, Hiroki Mori, Nicole Parker, Katy Hulland, Dominique Westbrook, Umar Burki, Jenny Hincks, Tony White, Vittorio Spampinato, Helen Bright, Steve Evans

**Affiliations:** 1 Inflammation and Remodeling Research, Pfizer Cambridge Massachusetts, 02140, USA

## 

Influenza A virus is one of the most common infectious pathogens. The disease that ensues is particularly serious in certain at risk groups within the population; such as individuals with chronic respiratory and cardiovascular diseases. Asthma is an example of a chronic inflammatory disease which is exacerbated by the virus, and infection frequently leads to hospitalization of asthmatic patients. The role of respiratory viruses in development of severe clinical asthma, in particular, is poorly understood. Pfizer has supported the European Union-led IMI UBIOPRED pre-competitive collaboration between academic and pharma partners which aims to better understand the clinical disease, and in doing so, developed pre-clinical models of both severe asthma and asthma exacerbation. We established and characterised RSV and Influenza A infection in the mouse house dust mite (HDM) model.

We have examined the bronchoalveolar fluid (bal) cell and lung cytokine responses as well as observing the health of the animals for a 14 day period following exposure to varying virus doses. Groups of mice were infected with 5 x virus doses and virus replication and inflammatory responses monitored at days 1, 3, 7 and 14 post infection. Bronchoalveolar lavage (BAL) and lungs were collected from all mice. BAL was processed for cell counts and tissue was processed for cytokine and pathology. Additionally, mice were observed daily for onset of clinical symptoms and weight loss. We have demonstrated two unique viral exacerbation profiles and demonstrated that influenza can generate an exacerbative profile in conjunction with HDM challenge.

